# Cytokine production capacity in depression and anxiety

**DOI:** 10.1038/tp.2016.92

**Published:** 2016-05-31

**Authors:** N Vogelzangs, P de Jonge, J H Smit, S Bahn, B W Penninx

**Affiliations:** 1 Department of Epidemiology, Cardiovascular Research Institute Maastricht & Maastricht Centre for Systems Biology, Maastricht University, Maastricht, The Netherlands; 2 Department of Psychiatry and EMGO Institute for Health and Care Research, VU University Medical Center, Amsterdam, The Netherlands; 3 Interdisciplinary Center Psychopathology and Emotion Regulation, Department of Psychiatry, University Medical Center Groningen, Groningen, University of Groningen, Groningen, The Netherlands; 4 Department of Chemical Engineering and Biotechnology, Institute of Biotechnology, University of Cambridge, Cambridge, UK

## Abstract

Recent studies have suggested that immune function may be dysregulated in persons with depressive and anxiety disorders. Few studies examined the expression of cytokines in response to *ex vivo* stimulation of blood by lipopolysaccharide (LPS) to study the innate production capacity of cytokines in depression and anxiety. To investigate this, baseline data from the Netherlands Study of Depression and Anxiety (NESDA) were used, including persons (18–65 years; 66% women) with current (that is, past month; *N*=591) or remitted (*N*=354) DSM-IV depressive or anxiety disorders and healthy controls (*N*=297). Depressive and anxiety symptoms were measured by means of the Inventory of Depressive Symptomatology (IDS) and the Beck Anxiety Inventory (BAI). Using Multi-Analyte Profiling technology, plasma levels of 13 cytokines were assayed after whole blood stimulation by addition of LPS. Basal plasma levels of C-reactive protein, interleukin-6 and tumor necrosis factor-α were also available. A basal and a LPS summary index were created. Results show that LPS-stimulated inflammation was associated with increased odds of current depressive/anxiety disorders (odds ratio (OR)=1.28, *P*=0.009), as was the case for basal inflammation (OR=1.28, *P*=0.001). These associations were no longer significant after adjustment for lifestyle and health (OR=1.13, *P*=0.21; OR=1.07, *P*=0.45, respectively). After adjustment for lifestyle and health, interleukin-8 was associated with both remitted (OR=1.25, *P*=0.02) and current (OR=1.28, *P*=0.005) disorders. In addition, LPS-stimulated inflammation was associated with more severe depressive (*β*=0.129, *P*<0.001) and anxiety (*β*=0.165, *P*<0.001) symptoms, as was basal inflammation. Unlike basal inflammation, LPS-stimulated inflammation was still associated with (anxiety) symptom severity after adjustment for lifestyle and health (IDS: IL-8, MCP-1, MMP2; BAI: LPS index, IL-6, IL-8, IL-10, IL-18, MCP-1, MMP2, TNF-β). To conclude, lifestyle and health factors may partly explain higher levels of basal, as well as LPS-stimulated inflammation in persons with depressive and anxiety disorders. However, production capacity of several cytokines was positively associated with severity of depressive and in particular anxiety symptoms, even while taking lifestyle and health factors into account. Elevated IL-8 production capacity in both previously and currently depressed and anxious persons might indicate a genetic vulnerability for these disorders.

## Introduction

Depression, as well as anxiety disorders are of major importance for public health.^1, 2, 3^ These disorders are associated with a great loss in quality of life,^4^ increased (cardiovascular) morbidity and mortality,^5, 6, 7^ and show a high rate of recurrence and chronicity.^8, 9, 10^ Treatment of depression and anxiety disorders, including the prevention of relapse, is only effective in about a third to a half of patients.^11, 12^ Nearly all available antidepressants, which are also often prescribed for anxiety disorders, act by increasing monoaminergic transmission, although the etiology of depression and anxiety is much more diverse. Extensive knowledge of other pathophysiological mechanisms is needed to guide development of new treatments for depression and anxiety disorders.

In the past decade, an increasing number of studies—as summarized in meta-analyses—has indicated that inflammatory marker levels such as C-reactive protein (CRP), interleukin (IL)-6 and tumor necrosis factor (TNF)-α are elevated in depressed persons signifying immune dysregulation.^13, 14^ Depression could bring about disturbances in important stress systems of the human body, that is, the hypothalamus–pituitary–adrenal axis^15, 16^ and the autonomic nervous system,^17, 18^ which in turn might stimulate production of cytokines.^19, 20^ On the other hand, administration of pro-inflammatory cytokines (for instance in cancer or hepatitis C treatment) has consistently been shown to induce depressive symptoms in about a third of patients.^21^

Depression shows a high degree of comorbidity with anxiety disorders,^22^ which suggests partly overlapping etiology. Some recent studies show evidence that immune dysregulation also plays a role in anxiety.^23, 24^ Although many studies have focused on depressive disorder or symptoms in general, increasing evidence suggests it is important to take clinical characteristics into account to delineate which depressed and anxious persons show dysregulation of the immune system. Previously, we found that in particular somatic symptoms of depression and anxiety, as compared with cognitive symptoms, were associated with inflammation.^25^ Also, we found inflammation levels to be elevated in atypical depression, but not in melancholic depression.^5, 26^ These findings are in line with the sickness behavior theory which argues that somatic depressive-like symptoms such as fatigue, sleeping problems, anorexia and motor slowing can be the result of upregulated inflammation levels.^27^

Although the theory of immune dysregulation in depression and possibly anxiety is appealing, not all evidence is consistent. One important aspect of earlier studies is that they have generally assessed basal circulating levels of inflammatory markers, which typically have low values, show a high degree of intra-individual variability and which might be strongly influenced by lifestyle and disease status. A meta-analysis by Howren *et al.*^14^ indicates that the association between depression and inflammation is largely weakened when taking body mass index (BMI) into account. The expression of inflammatory markers in response to *ex vivo* stimulation of blood by lipopolysaccharide (LPS) may give more insight into the functioning of immune regulation.^28^ It mimics the natural environment more closely and induces an inflammatory reaction reflecting the innate production capacity of inflammatory markers,^29^ which is known to be under strong genetic control.^30^ A recent study suggests that the variability seen when assessing basal levels, may be reduced by measuring inflammatory markers in LPS-stimulated blood, as LPS-stimulated, but not basal, inflammatory marker levels were found to be elevated in Alzheimer’s disease patients.^31^ With respect to depression, only one study showed that increased production of IL-1β and decreased IL-1 receptor antagonist production, as measured after LPS stimulation, preceded the development of depressive symptoms in a sample of older persons.^28^ So, although promising, up until now knowledge on whether LPS-stimulated cytokines, and which in particular, are related to depression and anxiety is rather lacking.

Considering all the above, the current study has four aims: to examine whether innate cytokine production capacity is associated with depressive and anxiety disorders (first aim) and symptom severity (second aim), also while considering the role of lifestyle and health factors. The third aim of this study is to investigate whether the innate cytokine production capacity is specifically associated with particular depressive and anxiety symptom subscales. We hypothesize to find specific associations with somatic, but not cognitive symptoms, and with atypical, but not melancholic symptoms. Finally, as basal levels of CRP, IL-6 and TNF-α are available, we are able to compare associations of basal versus LPS-stimulated cytokine levels with depression and anxiety within the same study sample (fourth aim). We expect more robust associations for the LPS-stimulated markers compared with the basal inflammation levels. We have no a priori hypothesis on which LPS-stimulated cytokines will be upregulated in depression and anxiety, but rather expect a general upregulation of cytokine production capacity.

## Materials and methods

### Sample

The Netherlands Study of Depression and Anxiety (NESDA) is an ongoing cohort study designed to investigate the long-term course and consequences of depressive and anxiety disorders. Participants were 18–65 years old at baseline assessment in 2004–2007 and were recruited from the community (19%), general practice (54%) and secondary mental health care (27%). To maintain representativity, only two exclusion criteria existed: (1) a primary clinical diagnosis of a psychiatric disorder not subject of NESDA, which will largely affect course trajectory: psychotic disorder, obsessive compulsive disorder, bipolar disorder or severe addiction disorder, and (2) not being fluent in Dutch since language problems would harm the validity and reliability of collected data. A total of 2981 persons were included, consisting of persons with a current or past depressive and/or anxiety disorder and healthy controls. A detailed description of the NESDA study design and sampling procedures can be found elsewhere.^32^ The research protocol was approved by the Ethical Committee of participating universities and after complete description of the study all respondents provided written informed consent.

Due to organizational reasons, LPS stimulation of blood was only conducted at the last year of the baseline sample collection. Consequently, data of LPS-stimulated inflammatory markers were available for a random sample of 1242 persons, which constitute the sample of the present study. Included persons did not differ from the remainder of NESDA respondents (*n*=1739) in terms of sex and years of education, but were somewhat older (42.8 (s.d.=12.7) vs 41.2 (s.d.=13.3) years, *P*=0.001) and had slightly more often no lifetime depressive or anxiety disorder (23.9% vs 20.4%, *P*=0.02).

### Depressive and anxiety disorders

During the baseline interview, lifetime presence of depressive disorder (major depressive disorder, dysthymia) and anxiety disorder (social phobia, generalized anxiety disorder, panic disorder, agoraphobia) was established using the Composite Interview Diagnostic Instrument (CIDI) according to DSM-IV criteria.^33^ The CIDI is a reliable and valid instrument for assessing depressive and anxiety disorders^34^ and was administered by specially trained research staff. Persons were grouped into ‘no depressive/anxiety disorder’, ‘remitted depressive/anxiety disorder’ (that is, lifetime presence, but not in the past month) and ‘current anxiety/depressive' disorder (that is, in the past month). Current disorders were further categorized into ‘depressive disorder only’, ‘anxiety disorder only’, and ‘comorbid depressive' and anxiety disorder’.

### Depressive and anxiety symptom severity

The severity of depressive symptoms was measured using the 30-item self-report Inventory of Depressive Symptomatology (IDS; range 0–84).^35^ The IDS has been shown to have good psychometric properties with high correlations between the IDS and the Hamilton Depression Rating Scale and the Beck Depression Inventory-I (www.ids-qids.org). The severity of anxiety symptoms was measured by means of the 21-item self-report Beck Anxiety Inventory (BAI; range 0-63).^36^ The internal and test–retest reliability and validity of the BAI are well-established.^36, 37^ Individual items on the IDS and BAI are rated from 0 to 3.

### Depressive and anxiety symptom subscales

As we have previously found basal inflammation to be more specifically associated with somatic symptoms than with cognitive symptoms,^25^ and more with atypical depression than with melancholic depression,^26^ we *a priori* created several symptom subscales. Previous research has shown that the BAI consists of two subscales accounting for 84% of the variance, that is, a somatic subscale and a cognitive subscale.^38^ The somatic subscale consists of 14 items (Cronbach’s *α*=0.90) and the cognitive subscale consists of 7 items (Cronbach’s *α*=0.88) (see Supplementary Table 1). A somatic and a cognitive symptoms subscale were also derived from the IDS, based on DSM-IV criteria and on previous research on somatic and cognitive depressive symptoms,^39, 40^ as used before.^25^ Both the somatic and the cognitive subscale consisted of 10 items (see Supplementary Table 1). Because the sleep symptoms were overrepresented, we created a variable combining the four sleep items by taking the mean score of all four items. This resulted in Cronbach’s *α*=0.79 for the somatic symptoms subscale and Cronbach’s *α*=0.90 for the cognitive symptoms subscale. Based on the IDS, we also constructed an atypical and a melancholic symptoms subscale, including all items corresponding to the DSM-IV criteria for atypical and melancholic depression (see Supplementary Table 1; atypical symptoms scale: Cronbach’s *α*=0.62; melancholic symptoms scale: Cronbach’s *α*=0.77).

### Inflammatory markers

Assaying of inflammatory markers was done by laboratory staff completely blinded to the clinical status of the participants. Markers of basal inflammation were assessed at baseline and included CRP, IL-6 and TNF-α. Fasting blood samples of NESDA participants were obtained in the morning around 0800 hours and kept frozen at −80 °C. CRP and IL-6 were assayed at the Clinical Chemistry department of the VU University Medical Center. High-sensitivity plasma levels of CRP were measured in duplicate by an in-house enzyme-linked immunosorbent assay (ELISA) based on purified protein and polyclonal anti-CRP antibodies (Dako, Glostrup, Denmark). Plasma IL-6 levels were measured in duplicate by a high-sensitivity ELISA (PeliKine Compact ELISA, Sanquin, Amsterdam, the Netherlands). Plasma TNF-α levels were assayed in duplicate at Good Biomarker Science, Leiden, the Netherlands, using a high-sensitivity solid phase ELISA (Quantikine HS Human TNF-α Immunoassay, R&D systems, Minneapolis, MN, USA). Intra- and inter-assay coefficients of variation were 5 and 10% for CRP, 8 and 12% for IL-6, and 10 and 15% for TNF-α. Distributions of CRP, IL-6 and TNF-α values were skewed to the right and therefore were ln-transformed to successfully normalize distributions.

To reflect the innate production capacity of inflammatory markers, the innate immune response of 17 cytokines was assessed in blood that was *ex vivo* stimulated with LPS. Serial venous whole blood samples were obtained at baseline in one 7-ml heparin-coated tube (Greiner Bio-one, Monroe, NC, USA). Between 10 and 60 min after blood draw, 2.5 ml of blood was transferred into a PAXgen tube (Qiagen, Valencia, CA, USA). Remaining blood (4.5 ml) was stimulated by addition of LPS (10 ng ml^−1^ blood; *Escherichia coli*, Sigma, St. Louis, MO, USA), as done by others.^28, 31^ LPS-stimulated samples were laid flat and incubated at a slow rotation for 5–6 h at 37 °C. A 2.5-ml sample of this LPS-stimulated blood was transferred into a PAXgene tube. This LPS procedure was carried out at four laboratories (Amsterdam, Leiden, Groningen, Heerenveen). Remaining plasma (±0.5 ml) was kept frozen at −80 °C for later assaying. Levels of granulocyte-macrophage colony-stimulating factor (GM-CSF), interferon (IFN)-γ, IL-2, IL-3, IL-4, IL-5, IL-6, IL-7, IL-8, IL-10, IL-18, monocyte chemotactic protein-1 (MCP-1), macrophage inflammatory protein (MIP)-1α, MIP-1β, matrix metalloproteinase-2 (MMP2), TNF-α and TNF-β were assayed simultaneously for all available samples, using a multi-analyte profile (Human CytokineMAP A v 1.0; Myriad RBM, Austin, TX, USA). This commercial platform adheres to stringent guidelines of quality control and has Clinical Laboratory Improvement Amendments (CLIA) approval, which means that the platform is validated and calibrated on a continuous basis.^41^ For GM-CSF, IL-3, IL-5 and IL-7 too little valid values were obtained (valid *n*<200) and were therefore excluded from further analyses, leaving a total of 13 cytokines. With the exception of MMP2 and TNF-β (which showed a normal distribution), all cytokines were skewed to the right and therefore were ln-transformed to successfully normalize distributions.

As we did not have specific hypotheses about individual inflammation markers, and rather sought to examine a general increased level of cytokine production capacity, we created an LPS-stimulated inflammation index, as well as a basal inflammation index. This also allowed us to better compare basal inflammation versus LPS-stimulated inflammation and to reduce the number of main analyses. A basal inflammation index was calculated as the standardized sum of all 3 ln-transformed (that is, normally distributed) standardized basal inflammation markers and an LPS-stimulated inflammation index was calculated as the standardized sum of all 13 (normally distributed) standardized LPS-stimulated inflammation markers. Standardization was based on the grand mean (s.d.) of each marker (that is, *N*=1242). Both inflammation indexes are considered the main inflammation variables of interest. For the most important analyses, individual inflammation markers are also analyzed and presented in a supplement to test consistency across markers.

### Covariates

Sociodemographic characteristics included sex and age. Several lifestyle and health characteristics were assessed as these can be associated with both psychopathology and inflammation. Smoking status (never, former, current) was self-reported. Alcohol intake was classified as <1, 1–14/1–21 (women/men) and >14/21 (women/men) drinks per week; based on general guidelines that are used in health organizations in the Netherlands^42^ and as used in other studies.^43^ Physical activity was measured with the International Physical Activity Questionnaire^44^ in MET-minutes (ratio of energy expenditure during activity compared with rest times the number of minutes performing the activity) per week. Body mass index was calculated as weight in kilograms divided by height in meters squared. The number of self-reported chronic diseases for which persons received treatment (including heart disease, diabetes, stroke, lung disease, osteoarthritis or rheumatic disease, cancer, ulcer, intestinal problem, liver disease, epilepsy and thyroid gland disease) was counted. Medication use was assessed based on drug container inspection of all drugs used in the past month and classified according to the World Health Organization Anatomical Therapeutic Chemical classification.^45^ Use of systemic anti-inflammatory medication included ATC codes M01A, M01B, A07EB and A07EC. In addition, regular antidepressant medication use included selective serotonin reuptake inhibitors (N06AB), serotonin–norepinephrine reuptake inhibitors (N06AX16, N06AX21), tricyclic antidepressants (N06AA) and tetracyclic antidepressants (N06AX03, N06AX05, N06AX11).

### Statistical analyses

Baseline characteristics across disorder status (no, remitted, current) were compared using *χ*^2^-tests for dichotomous and categorical variables and one-way analysis of variance (ANOVA) for normally distributed continuous variables. For descriptive purposes, all inflammatory markers are presented untransformed, though the *P*-values are based on one-way ANOVA of the normally distributed ln-transformed values. Pearson *r* correlations were estimated between all individual inflammatory markers and the basal and LPS-stimulated inflammation indexes. Associations of covariates with the basal inflammation index and the LPS-stimulated inflammation index were assessed using one-way ANOVA for dichotomous and categorical variables and Pearson *r* correlations for continuous variables. Associations of inflammation with depressive/anxiety disorder status and symptom severity were tested using multinomial logistic and linear regression analyses, adjusted for laboratory site, sex and age. Lifestyle and health factors may be part of the mechanism relating inflammation to depression/anxiety, which might especially be the case for BMI and chronic diseases, but could also be genuine confounding factors. Therefore, to avoid over-correction, but to still be able to check whether found associations are dependent on lifestyle and health characteristics, associations were additionally adjusted for smoking and alcohol intake and in a separate step additionally for BMI and number of chronic diseases. Last, linear regression analyses were performed assessing the association between the basal inflammation index and the LPS-stimulated inflammation index with the different symptom subscales (IDS somatic, IDS cognitive, BAI somatic, BAI cognitive, IDS atypical, IDS melancholic).

## Results

### Sample description

Mean age of the study sample was 42.8 (s.d.=12.7) years and 65.7% were women. Table 1 compares baseline characteristics across persons without a depressive or anxiety disorder (*n*=297), with a history of an anxiety or depressive disorder (*n*=354) and with a current (that is, in the past month) depressive or anxiety disorder (*n*=591; including 536 persons fulfilling complete diagnostic criteria in the past 2 weeks). Over the past 4.5 years, persons with a current disorder reported symptoms 58% (s.d.=35) of the time, corresponding to a median of 30 (interquartile range (IQR)=14–52) months. From the 354 remitted persons, 103 remitted from their depressive/anxiety disorder between 1 and 6 months ago, 28 between 6 months and 1 year ago, and 223 more than 1 year ago. Persons with a remitted disorder were somewhat older compared with the other groups. Persons with a current disorder were more often non-drinker, less physically active and had more chronic diseases than persons without depressive/anxiety disorder. Persons with a remitted or current disorder were also more often current smoker, tended to have a higher BMI and more often used anti-inflammatory medication compared with controls. Most basal, as well as LPS-stimulated inflammatory levels were highest among those with a current disorder.

### Inflammatory marker correlates

Pearson’s correlations (see Supplementary Table 2) between basal inflammatory markers were small (0.1–0.3), while LPS-stimulated inflammatory markers in general correlated much stronger (0.4–0.9, with some exceptions for IL-4 and IL-10). These moderate to strong correlations underline our strategy of adding standardized values to obtain the LPS-stimulated inflammation index. The Pearson’s correlation between the basal inflammation index and the LPS-stimulated inflammation index was small (*r*=0.13, *P*<0.001), suggesting that these are two distinct indicators of immune regulation. The associations of covariates with the basal and LPS-stimulated inflammation indexes are shown in Table 2. Sex was associated with the LPS-stimulated inflammation index (higher in men), but not the basal inflammation index, while alcohol intake, anti-inflammatory medication use and antidepressant medication use were positively associated with the basal inflammation index only. Higher age and current smoking similarly increased basal and LPS-stimulated inflammatory levels, while the size of association of BMI and the number of chronic disease was somewhat stronger with the basal inflammation index than with the LPS-stimulated inflammation index. Physical activity was associated with neither index.

### Inflammation and depressive/anxiety disorders

Table 3 shows the associations between the basal and LPS-stimulated inflammation indexes with remitted and current depressive and anxiety disorders after adjustment for site, sex and age. The basal inflammation index, as well as the LPS-stimulated inflammation index were not associated with a remitted disorder, but significantly increased the odds of a current depressive/anxiety disorder. The ORs of both the basal and the LPS-stimulated inflammation index were of similar size (both OR=1.28), also when including both indexes in one model (OR=1.25 and OR=1.20, respectively), suggesting independent effects of basal inflammation levels and LPS-stimulated inflammation levels on disorder status.

When excluding all persons with a chronic disease (that is, heart disease, diabetes, lung disease, osteoarthritis or rheumatic disease, cancer, intestinal problems; *n* excluded=260), results remained similar (separate models: basal index-current disorder: OR=1.30; LPS index-current disorder: OR=1.24). When adjusting the basic analyses on disorder status for depressive and anxiety symptoms (IDS and BAI, respectively), ORs decreased somewhat and were no longer significant, suggesting there is no independent effect of disorder status apart from symptom severity. To assess possible differential effects for depressive versus anxiety disorders, current disorders were stratified into pure depressive disorder (*n*=130), pure anxiety disorder (*n*=219) and comorbid disorder (*n*=242) and compared with controls in one multinomial logistic regression analysis, but results were rather similar across these three categories (basal index: OR=1.26, OR=1.18, OR=1.38; LPS index: OR=1.33, OR=1.26, OR=1.27).

Table 3 also presents results after additional adjustment for lifestyle (current smoking, alcohol intake) and further health factors (BMI, number of chronic diseases). After these adjustments, both indexes were no longer significantly associated with a current disorder.

Associations of individual inflammation markers with depressive/anxiety disorder are shown in Supplementary Table 3. After basic adjustment, higher basal levels of CRP and IL-6 were associated with the presence of current depressive/anxiety disorder. Also, several individual LPS-stimulated inflammation markers showed an increased OR for current disorder, which was significant for IL-8, IL-18, MCP-1, MIP-1β, MMP2 and TNF-β. IL-4 and IL-8 were (also) associated with a remitted depressive/anxiety disorder. To assess whether associations were independent of lifestyle and health factors, significant markers were additionally adjusted for current smoking, alcohol intake, BMI and number of chronic diseases (results not tabulated). Associations turned to non-significant, except for IL-8, which was still significantly associated with both remitted (OR=1.25, 95%CI=1.04–1.49, *P*=0.02) and current (OR=1.28, 95%CI=1.08–1.52, *P*=0.005) depressive/anxiety disorders. This finding remained similar after additional adjustment for physical activity and anti-inflammatory medication.

### Inflammation and depressive/anxiety symptom severity

Table 3 also shows the associations between the inflammation indexes and depressive (IDS) and anxiety (BAI) symptom severity after adjustment for site, sex and age. The basal inflammation index, as well as the LPS-stimulated inflammation index were significantly associated with both depressive and anxiety symptom severity. Even when including both indexes in one model, the basal and LPS-stimulated inflammation indexes both remained significant and were rather comparable in effect size for depressive symptom severity (*β*=0.122 and *β*=0.103, respectively), suggesting independent associations. For anxiety severity, when including the basal and LPS-stimulated inflammation indexes in the same model, the association of the LPS-stimulated inflammation index was somewhat larger than the association of the basal inflammation index (*β*=0.145 and *β*=0.091, respectively), although both were significant.

When excluding all persons with a chronic disease (that is, heart disease, diabetes, lung disease, osteoarthritis or rheumatic disease, cancer, intestinal problems; *n* excluded=260), associations decreased somewhat but were still significant (basal index: IDS: *β*=0.098, *P*=0.003; BAI: *β*=0.071, *P*=0.03; LPS index: IDS: *β*=0.108, *P*=0.01; BAI: *β*=0.134, *P*=0.001). Also, when additionally adjusting the basic analyses for depressive/anxiety disorder status, associations decreased somewhat but were still significant (basal index: IDS: *β*=0.067, *P*=0.002; BAI: *β*=0.052, *P*=0.03; LPS index: IDS: *β*=0.063, *P*=0.02; BAI: *β*=0.109, *P*<0.001). Due to a high correlation between IDS and BAI (Pearson’s *r*=0.80), depressive and anxiety symptom analyses could not be adjusted for each other.

Table 3 also presents results after additional adjustment for lifestyle (current smoking, alcohol intake) and further health factors (BMI, number of chronic diseases). Both the basal and LPS-stimulated inflammation indexes were associated with depressive symptom severity after lifestyle adjustment, but no longer after further health adjustment. In contrast, the basal inflammation index was already no longer associated with anxiety symptoms after lifestyle adjustment, while the LPS-stimulated inflammation index was still significantly associated with anxiety symptoms after both lifestyle and further health adjustment (*β*=0.099, *P*=0.005). This finding remained similar after additional adjustment for physical activity and anti-inflammatory medication.

Associations of individual inflammation markers with symptom severity are shown in Supplementary Table 3. Basal CRP, IL-6 and TNF-α, as well as most individual LPS-stimulated inflammation markers (IL-6, IL-8, IL-10, IL-18, MCP-1, MIP-1α, MIP-1β, MMP2 and TNF-β) were significantly associated with depressive and anxiety symptom severity. To check whether found associations were dependent on lifestyle and health factors, analyses were additionally adjusted for current smoking, alcohol intake, BMI and number of chronic diseases (results not tabulated). No associations remained for the basal inflammation markers. However, with respect to LPS-stimulated inflammation markers, associations remained significant for IL-8 (*β*=0.075, *P*=0.01), MCP-1 (*β*=0.097, *P*=0.003) and MMP2 (*β*=0.083, *P*=0.008) with depressive symptom severity, and for IL-6 (*β*=0.081, *P*=0.02), IL-8 (*β*=0.073, *P*=0.02), IL-10 (*β*=0.083, *P*=0.02), IL-18 (*β*=0.067, *P*=0.03), MCP-1 (*β*=0.099, *P*=0.003), MMP2 (*β*=0.124, *P*<0.001) and TNF-β (*β*=0.105, *P*=0.001) with anxiety symptom severity. Results remained similar after additional adjustment for physical activity and anti-inflammatory medication.

### Inflammation and depressive/anxiety symptom subscales

Table 4 shows associations between the basal and LPS-stimulated inflammation indexes and several depressive and anxiety symptom subscales. After basic adjustment, both indexes were significantly associated with all subscales. After lifestyle adjustment (current smoking, alcohol intake), in line with our earlier findings,^25, 26^ the basal inflammation index was associated with somatic depressive, somatic anxiety and atypical symptoms, but not with cognitive depressive, cognitive anxiety and melancholic symptoms. In contrast, the LPS-stimulated inflammation index was associated with somatic, as well as with cognitive depressive and anxiety symptoms. Also, after lifestyle adjustment, the association between the LPS-stimulated inflammation index with atypical and melancholic symptoms was of equal size, although statistically significant for the former and only a trend finding for the latter (*β*=0.074, *P*=0.04; *β*=0.069, *P*=0.06, respectively). After further adjustment for BMI and number of chronic diseases, associations weakened and were no longer significant for the basal inflammation index. The LPS-stimulated inflammation index was still significantly associated with somatic (*β*=0.098, *P*=0.005), as well as cognitive (*β*=0.085, *P*=0.02) anxiety symptoms.

## Discussion

This study examined innate cytokine production capacity in a large sample of persons with varying levels of depression and anxiety. Lifestyle and health factors may partly explain higher levels of basal, as well as LPS-stimulated inflammation in persons with depressive and anxiety disorders. However, we found that cytokine production capacity was positively associated with severity of depressive and in particular anxiety symptoms, even while taking lifestyle and health factors into account. Elevated IL-8 production capacity in both previously and currently depressed and anxious persons might indicate a genetic vulnerability for these disorders.

In the present study, we were able to compare associations of basal inflammation and cytokine production capacity in relation to depression and anxiety within the same study sample. Our results underline the idea that basal inflammation levels and stimulated inflammation levels tap on two different aspects of the immune system. The correlation between both concepts was rather low and associations of basal and stimulated levels with depression/anxiety seemed rather independent of each other, shown when including both inflammation indexes in one model. Possibly, part of this low correlation may be explained by the use of two different distinct methods that were used to assess the basal (ELISA) versus LPS-stimulated (multiplex) inflammation markers. However, we also found differences in associations with covariates. Basal inflammation appeared to be more strongly correlated to several lifestyle and health factors, such as alcohol intake, BMI, chronic diseases and medication use. In line with this, we found that associations between basal inflammation and depression/anxiety largely disappeared after adjustment for lifestyle and health factors. Although associations between LPS-stimulated inflammation levels and depression/anxiety also weakened after lifestyle and health adjustment, especially with respect to disorder status, several associations still remained (that is, IL-8 with remitted and current disorder, IL-8, MCP-1 and MMP2 with depressive symptom severity, and LPS-stimulated inflammation index, IL-6, IL-8, IL-10, IL-18, MCP-1, MMP2 and TNF-β with anxiety symptom severity). Prior research suggests a large role for BMI in the association between inflammation and depression.^14^ Our results suggest that this may even be more true for basal inflammation levels, which are highly variable. Furthermore, we and others have previously shown that antidepressant use might affect basal inflammation levels.^46, 47, 48^ Our current findings suggest that LPS-stimulated inflammation levels are less influenced by antidepressant use.

Our study found no difference in associations of the innate cytokine production capacity with depressive disorder versus anxiety disorder. Our results also showed associations of cytokine production capacity with both depressive and anxiety symptoms, before and after adjustment for current smoking and alcohol intake. However, after further adjustment for BMI and number of chronic diseases, LPS-stimulated inflammation was more consistently associated with anxiety symptoms (index and IL-6, IL-8, IL-10, IL-18, MCP-1, MMP2, TNF-β) than with depressive symptoms (IL-8, MCP-1, MMP2, but not the index). The relationship between inflammation and anxiety has been much less studied than the relationship with depression, but our results might suggest that cytokine production capacity, rather than basal inflammation is involved in anxiety. Future research should further investigate these explorative findings.

Innate cytokine production capacity was equally associated with cognitive and with somatic symptoms of depression and anxiety. Similarly, cytokine production capacity was to the same degree associated with melancholic and with atypical symptoms. In contrast, after lifestyle adjustment basal inflammation levels, in line with previous research,^5, 25, 26^ were in particular associated with somatic symptoms and with atypical symptoms, but not with cognitive and melancholic symptoms. This again underlines the differences between the two inflammation measures and suggests that innate cytokine production capacity might be generally involved in depression and anxiety, while basal inflammation is particularly important for depression and anxiety in which somatic health plays an important role. Of note is that one of the most important aspects of atypical depression is increased weight and appetite which would clearly affect somatic health as well. Interestingly, after additional adjustment for BMI and chronic diseases, the differential association of basal inflammation with somatic/atypical versus cognitive/melancholic symptoms disappeared.

Although we found significant associations for several specific LPS-stimulated inflammation markers with depression and in particular anxiety severity, the overall trend was increased levels of all markers, as also indicated by the LPS-stimulated inflammation index. At a closer look, this means that not only pro-inflammatory cytokines were positively associated with symptom severity, but also IL-10, which is an anti-inflammatory cytokine. This suggests that it is exactly the innate cytokine production capacity which is increased in depression and anxiety, regardless whether the response is pro- or anti-inflammatory. This is in contrast, however, with the study by van den Biggelaar,^28^ which found no association between IL-10 and the onset of depressive symptoms, and found a negative association between IL-1 receptor antagonist, also an anti-inflammatory agent, and the onset of depressive symptoms. Possibly, differences in design (cross-sectional versus longitudinal) and in sample (adults versus elderly) can explain these differences.

Innate cytokine production capacity was associated with both disorder status and symptom severity after basic adjustment. Due to the high overlap between these constructs (that is, current depressed/anxious persons score higher on IDS and BAI scales), it is difficult to determine independency of these associations. When adjusting disorder status for symptom severity, significances of associations were lost. On the other hand, the LPS-stimulated inflammation index was still significantly associated with both depression and anxiety severity after adjustment for disorder status. Furthermore, more individual markers were associated with (anxiety) severity than with disorder status and, unlike disorder status, most associations with (anxiety) severity remained significant after lifestyle and health adjustment. It is, however, too tentative to conclude whether innate cytokine production capacity is an underlying trait which linearly increases the risk for having more depressive and anxiety symptoms or whether it is especially associated with disorder status. In particular, IL-8 was profoundly associated with disorder status, in the same degree to remitted as to current disorders, even after adjustment for several lifestyle and health factors. This might suggest a possible (genetic) vulnerability and is in line with previous findings showing that maternal IL-8 increases schizophrenia risk in offspring.^49^ Future research should further investigate the role of IL-8 in depression and anxiety.

Some limitations of our study need to be acknowledged. First, our study had a cross-sectional design, which makes it impossible to draw any conclusions about causality. However, the innate cytokine production capacity, as measured via LPS-stimulated inflammation levels, has been shown to be under strong genetic control. Heritability estimates of the production capacity of various cytokines range from 53 to 86% in non-diseased populations.^30, 50^ This could indicate that cytokine production capacity is more likely to be a causal factor than the consequence of depression or anxiety. However, longitudinal studies are needed to investigate this more thoroughly. Another limitation is that data on the innate cytokine production capacity were only available in about 40% of the total NESDA sample. However, the sample size was still quite large and the sample was random. Included participants were rather comparable on baseline characteristics compared with the remainder of the NESDA participants. Furthermore, since we excluded persons with severe mental disorders, our results might not be generalizable to the subgroup of depressed and anxious persons which have a severe comorbid mental disorder. Specifically the exclusion of bipolar disorder could have impacted our results. Bipolar disorder has also been associated with inflammation.^51, 52^ Our results show that depression and anxiety are associated with inflammation even without the presence of bipolar disorder. Furthermore, since persons with depressive and anxiety disorders across different settings and developmental stages were included, our result should be generalizable to the greater part of depressed and anxious persons. Another limitation is that symptom severity was based on self-report and not clinically rated. Last, due to the high correlations between depressive and anxiety symptoms and between disorder status and symptom severity, we were not able to include these outcomes in a single analysis to directly compare the magnitude of associations of inflammation with these factors. Nonetheless, our study also had some important strengths. This study was among the first to assess the association between LPS-stimulated inflammation levels and depression and anxiety. Furthermore, we were able to compare our findings for innate cytokine production capacity with those for basal inflammation levels within the same study sample, although these comparisons have to be made with caution because of the methodological differences and differences in type and number of inflammatory markers between these two measures. Our study provides clear evidence that this novel technique of *ex vivo* stimulation is useful in detecting immune dysregulation in depression and anxiety. Another strong aspect of this study was the possibility to control for several lifestyle and health factors that were accurately examined.

In conclusion, lifestyle and health factors may partly explain higher levels of basal, as well as LPS-stimulated inflammation in persons with depressive and anxiety disorders. However, innate cytokine production capacity is positively associated with severity of depressive and in particular anxiety symptoms, even while taking lifestyle and health factors into account. IL-8 production capacity might indicate a genetic vulnerability for depressive and anxiety disorders. Measuring innate immune activity gives additional insight into the role of the immune system in depression and anxiety.

## Figures and Tables

**Table 1:** Baseline characteristics according to depressive/anxiety disorder status

	*No* *depressive or anxiety disorder* N*=297*	*Remitted depressive or anxiety disorder* N*=354*	*Current depressive or anxiety disorder* N*=591*	P^a^
*Demographics, lifestyle and health*				
Women, %	62.0	66.4	67.2	0.29
Age (years), mean (s.d.)	42.2 (13.9)	44.2 (12.5)	42.2 (12.1)	0.04
Smoking status, %				<0.001
Never smoker	37.7	26.0	26.2	
Former smoker	38.7	36.7	29.9	
Current smoker	23.6	37.3	43.8	
Alcohol intake, %				<0.001
None	21.2	25.4	40.1	
Moderate	66.7	64.4	47.5	
Heavy	12.1	10.2	12.4	
Physical activity (1000 MET-min), mean (s.d.)	4.0 (3.1)	3.8 (2.9)	3.5 (3.0)	0.04
Body mass index, mean (s.d.)	25.2 (4.7)	25.7 (4.8)	25.9 (5.3)	0.14
Number of chronic diseases, mean (s.d.)	0.5 (0.7)	0.6 (0.8)	0.8 (1.0)	<0.001
Anti-inflammatory medication use, %	2.0	7.1	5.6	0.01
				
*Clinical characteristics*				
Current disorder status, %				NA
Pure depressive disorder	NA	NA	22.0	
Pure anxiety disorder	NA	NA	37.1	
Comorbid depressive/anxiety disorder	NA	NA	40.9	
Depression severity (IDS), mean (s.d.)	7.2 (6.8)	15.8 (10.0)	31.0 (12.5)	<0.001
IDS somatic score, mean (s.d.)	2.2 (2.3)	4.3 (3.1)	7.9 (3.9)	<0.001
IDS cognitive score, mean (s.d.)	1.7 (2.6)	5.0 (4.4)	11.7 (5.9)	<0.001
IDS atypical score, mean (s.d.)	1.3 (1.6)	2.6 (2.0)	4.7 (2.8)	<0.001
IDS melancholic score, mean (s.d.)	1.4 (2.2)	3.6 (3.6)	8.2 (4.6)	<0.001
Anxiety severity (BAI), mean (s.d.)	3.2 (4.4)	8.3 (7.3)	18.3 (10.8)	<0.001
BAI somatic score, mean (s.d.)	2.1 (3.0)	5.2 (5.1)	10.8 (7.3)	<0.001
BAI cognitive score, mean (s.d.)	1.1 (1.8)	3.1 (3.0)	7.5 (4.6)	<0.001
				
*Basal inflammatory markers*				
CRP (mg l^−1^), median (IQR)^b^	1.08 (0.51–2.37)	1.22 (0.54–2.74)	1.34 (0.60–3.47)	0.01
IL-6 (pg ml^−1^), median (IQR)^b^	0.71 (0.50–1.20)	0.73 (0.47–1.25)	0.85 (0.54–1.44)	0.01
TNF-α (pg ml^−1^), median (IQR)^b^	0.70 (0.60–1.10)	0.80 (0.60–1.10)	0.80 (0.60–1.10)	0.42
Basal inflammation index, mean (s.d.)	−0.13 (0.90)	−0.06 (1.00)	0.10 (1.03)	0.002
				
*LPS-stimulated inflammatory markers*				
IFN-γ (pg ml^−1^), median (IQR)^b^	9.8 (6.5–15.7)	9.7 (6.5–13.9)	10.2 (7.4–14.5)	0.15
IL-2 (pg ml^−1^), median (IQR)^b^	8.0 (5.6–11.5)	8.4 (5.6–12.2)	9.1 (6.2–13.3)	0.04
IL-4 (pg ml^−1^), median (IQR)^b^	8.2 (4.0–13.4)	9.0 (4.1–15.9)	9.0 (4.4–14.1)	0.13
IL-6 (ng ml^−1^), median (IQR)^b^	25.2 (15.5–35.8)	24.2 (15.9–33.5)	26.9 (18.3–36.1)	0.01
IL-8 (ng ml^−1^), median (IQR)^b^	9.1 (5.8–13.4)	10.5 (6.6–15.6)	11.1 (7.6–16.4)	<0.001
IL-10 (pg ml^−1^), median (IQR)^b^	205 (98–374)	200 (112–383)	206 (115–406)	0.12
IL-18 (pg ml^−1^), median (IQR)^b^	241 (193–286)	248 (196–298)	254 (210–317)	0.001
MCP-1 (ng ml^−1^), median (IQR)^b^	1.4 (0.9–2.1)	1.3 (0.8–2.2)	1.6 (1.1–2.4)	<0.001
MIP-1α (ng ml^−1^), median (IQR)^b^	17.2 (11.3–25.0)	17.0 (10.8–23.9)	18.6 (12.4–25.5)	0.006
MIP-1β (ng ml^−1^), median (IQR)^b^	219 (151–299)	228 (159–305)	241 (170–320)	0.003
MMP2 (ng ml^−1^), mean (s.d.)	69.4 (19.5)	70.4 (20.5)	74.1 (18.8)	0.001
TNF-α (ng ml^−1^), median (IQR)^b^	2.8 (1.8–4.2)	2.8 (1.8–4.1)	2.8 (1.9–4.1)	0.59
TNF-β (pg ml^−1^), mean (s.d.)	299 (135)	305 (140)	328 (133)	0.003
LPS-stimulated inflammation index, mean (s.d.)	−0.13 (1.06)	−0.07 (1.08)	0.11 (0.90)	0.001

Abbreviations: ANOVA, analysis of variance; BAI, Beck anxiety inventory; CRP, C-reactive protein; IDS, inventory of depressive symptomatology; IFN, interferon; IL, interleukin; LPS, lipopolysaccharide; MCP, monocyte chemotactic protein; MIP, macrophage inflammatory protein; MMP, matrix metalloproteinase; NA, not applicable; TNF, tumor necrosis factor.

a *P*-values based on *χ*^2^-test for dichotomous and categorical variables and one-way ANOVA for continuous variables.

b Median (IQR) is presented because variable is non-normally distributed; however, *P*-values are based on one-way ANOVA of the normally distributed ln-transformed values.

**Table 2:** Associations of baseline characteristics with inflammation indexes

		*Basal index*		*LPS index*	
	N	*Mean (s.d.) or* r	P^a^	*Mean (s.d.) or* r	P^a^
*Sex*			0.59		<0.001
Men	426	0.02 (1.00)		0.25 (1.03)	
Women	816	−0.01 (1.00)		−0.13 (0.96)	
					
Age	1242	0.16	<0.001	0.13	<0.001
					
*Smoking status*					
Never smoker	359	−0.05 (1.00)	Ref	−0.13 (1.00)	Ref
Former smoker	422	−0.11 (0.99)	0.36	−0.04 (0.98)	0.21
Current smoker	461	0.14 (1.00)	0.007	0.15 (1.00)	<0.001
					
*Alcohol intake*					
None	390	0.26 (1.06)	Ref	−0.02 (0.99)	Ref
Moderate	707	−0.15 (0.95)	<0.001	−0.01 (0.99)	0.85
Heavy	145	0.03 (0.92)	0.02	0.07 (1.08)	0.37
					
Physical activity	1242	−0.01	0.71	0.00	0.91
Body mass index	1242	0.40	<0.001	0.14	<0.001
Number of chronic diseases	1242	0.20	<0.001	0.13	<0.001
					
*Anti-inflammatory medication*			0.03		0.69
No	1178	−0.01 (1.00)		0.00 (1.01)	
Yes	64	0.27 (0.93)		0.05 (0.82)	
					
*Antidepressant medication*			<0.001		0.18
No	947	−0.07 (0.98)		−0.03 (1.01)	
SSRI	199	0.15 (1.05)		0.09 (0.97)	
SNRI	43	0.28 (0.90)		0.03 (1.04)	
TCA/TeCA	53	0.41 (1.06)		0.20 (0.79)	

Abbreviations: ANOVA, analysis of variance; SNRI, serotonin–norepinephrine reuptake inhibitor; SSRI, selective serotonin reuptake inhibitor; TCA, tricyclic antidepressant; TeCA, tetracyclic antidepressant.

a Based on one-way ANOVA for dichotomous and categorical variables and Pearson’s r for continuous variables.

**Table 3:** Associations^a^ of inflammation indexes with depressive/anxiety disorder and severity

		*Remitted disorder* *vs no disorder* N*=354* vs N*=297*			*Current disorder* vs *no disorder* N*=591* vs N*=297*			*Depression severity (IDS)* N*=1228*		*Anxiety* *severity (BAI)* N*=1230*	
	N	*OR*	*95%CI*	P	*OR*	*95%CI*	P	β	P	β	P
*Basal inflammation index*											
Basic^b^	1231	1.07	0.91–1.25	0.44	1.28	1.10–1.48	0.001	0.136	<0.001	0.111	<0.001
Lifestyle^c^	1231	1.01	0.85–1.19	0.94	1.12	0.96–1.31	0.15	0.071	0.01	0.047	0.10
Health^d^	1231	0.97	0.81–1.16	0.72	1.07	0.90–1.26	0.45	0.027	0.37	0.006	0.84
											
*LPS-stimulated inflammation index*											
Basic^b^	1241	1.12	0.92–1.36	0.27	1.28	1.06–1.54	0.009	0.129	<0.001	0.165	<0.001
Lifestyle^c^	1241	1.08	0.88–1.32	0.47	1.19	0.98–1.44	0.08	0.095	0.008	0.128	<0.001
Health^d^	1241	1.06	0.86–1.30	0.60	1.13	0.93–1.38	0.21	0.060	0.09	0.099	0.005
											
*Basal and LPS index in one model (basic adjustment)* b											
Basal	1231	1.05	0.90–1.24	0.53	1.25	1.08–1.45	0.004	0.122	<0.001	0.091	0.002
LPS	1231	1.10	0.90–1.35	0.44	1.20	0.99–1.45	0.06	0.103	0.006	0.145	<0.001

Abbreviations: BAI, Beck anxiety inventory; CI, confidence interval; IDS, inventory of depressive symptomatology; LPS, lipopolysaccharide; OR, odds ratio.

a Based on multinomial logistic regression analyses with disorder status as outcome (no lifetime disorder=reference group) and on linear regression analyses with severity as outcome.

b Basic=adjusted for site, sex and age.

c Lifestyle=basic+adjusted for current smoking and alcohol intake.

d Health=lifestyle+adjusted for body mass index and number of chronic diseases.

**Table 4:** Associations^a^ of inflammatory indexes with symptom subscales (*N*=1242)

	*IDS somatic*		*IDS cognitive*		*BAI somatic*		*BAI cognitive*		*IDS atypical*		*IDS melancholic*	
	β	P	β	P	β	P	β	P	β	P	β	P
*Basal inflammation index*												
Basic^b^	0.153	<0.001	0.094	0.001	0.127	<0.001	0.064	0.03	0.136	<0.001	0.090	0.002
Lifestyle^c^	0.095	0.001	0.036	0.22	0.067	0.02	0.008	0.77	0.086	0.003	0.033	0.26
Health^d^	0.037	0.22	−0.001	0.98	0.019	0.52	−0.015	0.63	0.010	0.75	0.010	0.75
												
*LPS-stimulated inflammation index*												
Basic^b^	0.131	<0.001	0.113	0.002	0.169	<0.001	0.132	<0.001	0.098	0.007	0.102	0.006
Lifestyle^c^	0.099	0.006	0.085	0.02	0.133	<0.001	0.101	0.005	0.074	0.04	0.069	0.06
Health^d^	0.057	0.11	0.059	0.10	0.098	0.005	0.085	0.02	0.025	0.47	0.049	0.18

Abbreviations: BAI, Beck anxiety inventory; IDS, inventory of depressive symptomatology; LPS, lipopolysaccharide

a Based on linear regression analyses with symptom profile scores as outcome.

b Basic=adjusted for site, sex and age.

c Lifestyle=basic+adjusted for current smoking and alcohol intake.

d Health=lifestyle+adjusted for body mass index and number of chronic diseases.
