# Supplementary material for: Cytokine production capacity in depression and anxiety
Source: Transl Psychiatry. 2016 May 31;6(5):e825–. doi: 10.1038/tp.2016.92 (PMC5070051; doi:10.1038/tp.2016.92)
Supplement: Supplementary Information [file tp201692x1.pdf]

Supplementary Table 1. Overview of items included in the different symptom subscales

IDS somatic (range 0-21)	IDS cognitive (range 0-30)	BAI somatic (range 0-42)	BAI cognitive (0-21)	IDS Atypical (range 0-15)	IDS melancholic (range 0-30)
1. Falling asleep[1]	5. Feeling sad	1. Numbness or tingling	4. Unable to relax	4. Sleeping too much	3. Waking up too early
2. Sleep during the night[1]	6. Feeling irritable	2. Feeling hot	5. Fear of worst happening	12. Increased appetite	8. Mood reactivity
3. Waking up too early[1]	10. Quality of your mood	3. Wobbliness in legs	9. Terrified or afraid	14. Increased weight	9. Mood worse in morning
4. Sleeping too much[1]	15. Concentration/ decision making	6. Dizzy or light-headed	10. Nervous	29. Interpersonal sensitivity	10. Quality of your mood
11/12. Appetite change	16. View of myself	7. Heart pounding/racing	14. Fear of losing control	30. Leaden paralysis	11. Decreased appetite
13/14. Weight change	18. Thoughts of death or suicide	8. Unsteady	16. Fear of dying		13. Decreased weight
20. Energy level	19. General interest	11. Feeling of choking	17. Scared		16. View of myself
23. Feeling slowed down	21. Capacity for pleasure	12. Hands trembling			21. Capacity for pleasure
24. Feeling restless	22. Interest in sex	13. Shaky/unsteady			23. Feeling slowed down
30. Leaden paralysis	29. Interpersonal sensitivity	15. Difficulty in breathing			24. Feeling restless
		18. Indigestion			
		19. Faint/light-headed			
		20. Face flushed			
		21. Hot/cold sweats			

IDS = Inventory of Depressive Symptomatology; BAI = Beck Anxiety Inventory.
[1] Merged into one variable reflecting sleep problems; numbers indicate item number on IDS or BAI.

Supplementary Table 2. Pearson r correlations between basal and LPS-stimulated inflammatory markers (N=1242)

	CRP	IL-6	TNF-α	Index	IFN-γ	IL-2	IL-4	IL-6	IL-8	IL-10	IL-18	MCP1	MIP-1α	MIP-1β	MMP2	TNF-α	TNF-β	Index
**Basal inflammatory markers**																		
CRP																		
IL-6	.32																	
TNF-α	.13	.09																
Index	.72	.70	.61															
**LPS-stimulated inflammatory markers**																		
IFN-γ	.00	-.04	.06	.01														
IL-2	.08	.02	.03	.06	.54													
IL-4	.05	.08	.02	.08	.26	.33												
IL-6	.08	-.02	.08	.06	.77	.61	.26											
IL-8	.18	.13	.02	.17	.31	.41	.32	.52										
IL-10	.05	-.02	.00	.01	.46	.26	.08	.49	.22									
IL-18	.22	.11	.15	.24	.42	.42	.28	.52	.50	.24								
MCP-1	.20	.14	.08	.21	.51	.44	.22	.63	.61	.47	.51							
MIP-1α	.08	.07	.09	.12	.66	.55	.30	.82	.65	.44	.52	.68						
MIP-1β	.10	.06	.10	.13	.67	.51	.23	.81	.52	.58	.49	.69	.92					
MMP2	.11	.07	.04	.11	.65	.66	.36	.77	.56	.49	.54	.75	.76	.75				
TNF-α	-.01	-.06	.10	.01	.77	.53	.26	.84	.40	.44	.48	.47	.77	.75	.66			
TNF-β	.07	-.04	.06	.04	.64	.67	.32	.73	.41	.48	.51	.52	.66	.67	.79	.67		
Index	.12	.05	.09	.13	.78	.71	.43	.90	.66	.58	.66	.77	.89	.88	.89	.82	.82	

CRP=C-Reactive Protein, IFN=Interferon, IL=Interleukin, LPS=lipopolysaccharide, MCP=Monocyte Chemotactic Protein, MIP=Macrophage Inflammatory Protein, MMP=Matrix Metalloproteinase, TNF=Tumor Necrosis Factor.
All r ≥ .06 have $p<.05$; all r ≥ .09 have $p<.001$.

Supplementary Table 3. Associations [a] of individual inflammatory markers [b] with depressive/anxiety disorder and severity

		Remitted disorder vs. no disorder N=354 vs. N=297			Current disorder vs. no disorder N=591 vs. N=297			Depression severity (IDS) N=1228		Anxiety severity (BAI) N=1230	
	N	OR	95%CI	p	OR	95%CI	p	β	p	β	p
Basal inflammatory markers											
CRP	1238	1.08	0.93-1.27	.31	1.22	1.06-1.41	.007	.094	.001	.073	.01
IL-6	1239	0.97	0.83-1.13	.67	1.19	1.03-1.38	.02	.105	<.001	.080	.005
TNF-α	1234	1.07	0.91-1.26	.40	1.10	0.95-1.27	.19	.070	.01	.064	.03
LPS-stimulated inflammatory markers											
IFN-γ	1242	0.86	0.72-1.04	.12	0.93	0.78-1.11	.44	-.006	.87	.047	.18
IL-2	1242	1.10	0.93-1.31	.26	1.10	0.94-1.29	.22	.043	.17	.053	.09
IL-4	1242	1.18	1.01-1.39	.04	1.10	0.95-1.27	.21	.038	.19	.008	.79
IL-6	1241	0.98	0.80-1.19	.83	1.11	0.92-1.33	.29	.071	.06	.124	.001
IL-8	1241	1.33	1.12-1.58	.001	1.44	1.23-1.70	<.001	.143	<.001	.144	<.001
IL-10	1242	1.03	0.85-1.26	.76	1.19	0.99-1.44	.07	.097	.01	.125	.001
IL-18	1242	1.17	0.99-1.39	.07	1.31	1.12-1.54	.001	.114	<.001	.135	<.001
MCP-1	1242	1.03	0.86-1.24	.75	1.28	1.08-1.51	.004	.170	<.001	.175	<.001
MIP-1α	1241	1.06	0.87-1.28	.57	1.14	0.96-1.37	.14	.061	.09	.092	.01
MIP-1β	1241	1.09	0.90-1.32	.38	1.25	1.05-1.49	.01	.101	.005	.130	<.001
MMP2	1242	1.08	0.90-1.30	.39	1.24	1.05-1.46	.01	.136	<.001	.175	<.001
TNF-α	1241	0.92	0.77-1.12	.41	0.95	0.79-1.13	.53	-.008	.83	.031	.37
TNF-β	1242	1.07	0.89-1.28	.48	1.21	1.03-1.42	.02	.093	.004	.142	<.001

CRP=C-Reactive Protein, IFN=Interferon, IL=Interleukin, LPS=lipopolysaccharide, MCP=Monocyte Chemotactic Protein, MIP=Macrophage Inflammatory Protein, MMP=Matrix Metalloproteinase, TNF=Tumor Necrosis Factor.

[a] Based on multinomial logistic regression analyses with disorder status as outcome (no lifetime disorder = reference group) and on linear regression analyses with severity as outcome, adjusted for site, sex, and age.

[b] All markers were standardized: (value – grand mean)/SD; because of non-normal distributions standardization was performed after ln-transformation to normalize distributions for all markers except MMP2 and TNF-β (which were already normally distributed).
